# Biomarkers for Early Detection of Postoperative Intra-Abdominal Abscess in Children with Perforated Appendicitis

**DOI:** 10.3390/jcm15197496

**Published:** 2026-09-26

**Authors:** Bianka Dujić, Sara Vuković, Jana Buzuk, Marko Bašković

**Affiliations:** 1 School of Medicine, Catholic University of Croatia, Ilica 242, 10000 Zagreb, Croatia; 2 Department of Pediatric Surgery, Children’s Hospital Zagreb, Ulica Vjekoslava Klaića 16, 10000 Zagreb, Croatia; 3 School of Medicine, University of Zagreb, Šalata 3, 10000 Zagreb, Croatia; 4 Scientific Centre of Excellence for Reproductive and Regenerative Medicine, School of Medicine, University of Zagreb, Šalata 3, 10000 Zagreb, Croatia; 5 Croatian Academy of Medical Sciences, Kaptol 15, 10000 Zagreb, Croatia

**Keywords:** biomarkers, complete blood count, perforated appendicitis, appendectomy, intraabdominal abscess, prognosis, children, pediatric surgery

## Abstract

**Background/Objectives**: Appendicitis is a common pediatric surgical emergency, with perforation leading to significant complications such as intra-abdominal abscesses. Current diagnostic methods are not sufficiently investigated in terms of timeliness, which emphasizes the need for reliable, non-invasive biomarkers for early signaling of postoperative abscess formation. This study aims to evaluate the associations between laboratory markers and postoperative intra-abdominal abscess formation in children treated for perforated appendicitis. **Methods**: This study retrospectively analyzed records of children treated for acute perforated appendicitis over ten years to identify differences in blood variables and clinical, radiological, and surgical factors between those who developed postoperative intra-abdominal abscesses and those who did not. **Results**: A total of 206 patients met the inclusion criteria. Of these, 30 developed an intra-abdominal abscess. Of the preoperative clinical factors, only rebound tenderness was significantly more present in the group that developed an abscess. Between the observed groups, leukocytes and neutrophils showed significant higher results in the group that developed an abscess at all observed time points, while C-reactive protein (CRP) showed a significant difference from the 4th postoperative day onwards. Neutrophil-to-lymphocyte ratio (NLR), neutrophil-to-monocyte ratio (NMR), monocyte-to-lymphocyte ratio (MLR), systemic immune-inflammation index (SII) and systemic inflammatory response index (SIRI) were significantly higher after 72 h, while SIRI showed a significant difference already after 24 h, and NLR and SII already within 24 h postoperatively. NLR and SIRI showed a significant mean percentage increase between samples taken on days 2 and 3 compared to samples taken on days 4 and 5. CRP showed a significantly smaller mean percentage decrease during this interval, as well as in the interval between samples taken on days 4 and 5, compared to samples taken on days 6 and 7. Looking at the preoperative variables of interest, neutrophil percentage was the only significant predictor. Because these serial postoperative comparisons were not corrected for multiple testing, they should be interpreted as exploratory rather than confirmatory. **Conclusions**: Blood biomarkers demonstrate significant postoperative changes within the early postoperative period that are associated with intra-abdominal abscess formation in children treated for perforated appendicitis.

## 1. Introduction

Appendicitis is the most common acute surgical condition in children, with perforation occurring in approximately 20–45% of cases, depending on demographic and healthcare system factors [1]. Perforated appendicitis remains a significant clinical challenge within pediatric surgical practice due to its associated complications and the complexity of postoperative management [2,3]. Perforation signifies a progression from simple inflammation to a complicated intra-abdominal process, often leading to localized or generalized peritonitis, abscess formation, and systemic inflammatory response syndrome (SIRS) [4]. The pathophysiology of perforated appendicitis involves transmural necrosis of the appendix, leading to direct bacterial contamination of the peritoneal cavity and subsequent abscess development [5]. Despite advances in diagnostic imaging and surgical techniques, accurately predicting which pediatric patients with perforated appendicitis are at increased risk of postoperative intra-abdominal abscess remains a significant clinical challenge. Current postoperative monitoring and diagnostics strategies primarily rely on clinical assessment and imaging modalities such as ultrasonography and computed tomography (CT), which, while useful, are limited by variability in sensitivity, specificity, and timeliness [2,6,7]. Therefore, there is a strong interest in discovering reliable, non-invasive biomarkers that could aid in assessing the risk of postoperative complications, particularly intra-abdominal abscess formation, to improve risk stratification and tailor postoperative care.

Laboratory indicators reflecting systemic inflammatory response, infection severity, and immune status have been extensively investigated in various infectious and inflammatory conditions, including appendicitis [8,9]. Blood count parameters are routinely measured in clinical practice. Several studies have suggested that elevated preoperative or postoperative levels of biomarkers might correlate with disease severity and complication risk in pediatric appendicitis [9,10,11,12]. However, the value of these laboratory indicators specifically for intra-abdominal abscess development following perforated appendicitis in children remains inadequately defined.

Given the heterogeneity of pediatric patients and the multifactorial nature of abscess formation, a comprehensive evaluation of laboratory markers, possibly combined with clinical and surgical parameters, could improve the ability to recognize patients at higher risk. A deeper understanding of the relationship between laboratory indicators and postoperative intra-abdominal abscess formation could facilitate earlier identification of higher-risk patients, prompt initiation of targeted interventions, and potentially reduce the incidence of postoperative complications. This approach aligns with the broader goals of personalized medicine and evidence-based surgical care, emphasizing the importance of biomarkers in improving clinical outcomes [13,14].

Despite advances in diagnostic modalities, the timely and reliable detection of postoperative abscesses remains elusive. Laboratory biomarkers offer a promising avenue for risk stratification, yet their utility warrants further rigorous investigation. This study aims to investigate the association between laboratory indicators and the development of intra-abdominal abscesses in pediatric patients undergoing surgery for perforated appendicitis, to provide insights that may optimize postoperative laboratory follow-up and improve prognoses in this vulnerable population.

## 2. Materials and Methods

### 2.1. Patients

For the purposes of the study, electronic patient records in the hospital information system (IN2 BIS^®^, *v216.0.000*, Constellation Software Inc., Toronto, ON, Canada) were retrospectively reviewed. The search included all patients who were surgically treated at the Children’s Hospital Zagreb with a diagnosis of K35 or its variants K35.0–K35.9 (Acute appendicitis—according to the International Classification of Diseases, 10th revision) in the ten-year period from 1 January 2016 to 1 January 2026. The inclusion criteria were all operated patients in whom the diagnosis of perforated appendicitis was confirmed intraoperatively and then histopathologically. The diagnosis of perforated appendicitis was confirmed intraoperatively by the pediatric surgeons, who documented evidence of transmural perforation. Cases with iatrogenic perforations were excluded to maintain consistency, as our focus was on spontaneous, full-thickness perforations validated by histopathology. The exclusion criteria were patients in whom a perityphlitic abscess was diagnosed intraoperatively, who had a COVID-19 infection at the time of surgery, who developed another viral or bacterial infection perioperatively, who developed a surgical wound infection, who had additional surgical pathology or in whom there was a lack of data for analysis (that is, insufficient perioperative clinical or laboratory documentation in the medical record to reliably construct the study variables). After identifying patients of interest, patients were divided into two groups depending on whether or not they developed an intra-abdominal abscess postoperatively. A flowchart of the study is shown in Figure 1.

### 2.2. Study Design

The following parameters were recorded for each patient: sex, age, the season in which the patient underwent surgery, time of surgery within the day, associated diseases, previous abdominal surgeries, preoperative antibiotic administration, duration of symptoms, right lower quadrant (RLQ) tenderness, nausea or vomiting, rebound tenderness, body temperature, preoperative ultrasound (US), appendix diameter measured by US, presence of a collection on US (localized peri-appendiceal fluid detected on imaging, which indicates an inflammatory or fluid-filled area near the appendix, but does not necessarily meet the criteria for a mature abscess), presence of fecalith on US, type of surgical procedure, need for surgical conversion, presence of drain, omentectomy performed, duration of surgery (time from the initial skin incision, or trocar insertion in the case of laparoscopic procedures, to the completion of wound closure), type of pathogen from the swab, total length of hospital stay (LOS), length of stay in the intensive care unit (ICU), type of antibiotics used, the need for antibiotic conversion and the reason for this. The following parameters were determined from the blood: erythrocytes, hemoglobin, hematocrit, mean corpuscular volume (MCV), mean corpuscular hemoglobin (MCH), mean corpuscular hemoglobin concentration (MCHC), red cell distribution width—coefficient of variation (RDW-CV), platelets, mean platelet volume (MPV), leukocytes, neutrophils (percentage, absolute), lymphocytes (percentage, absolute), monocytes (percentage, absolute), eosinophils (percentage, absolute), basophils (percentage, absolute), immature granulocytes (IG) (percentage, absolute), erythroblasts (percentage, absolute), C-reactive protein (CRP). Based on certain previous parameters, NLR (Neutrophil-to-Lymphocyte Ratio), NMR (Neutrophil-to-Monocyte Ratio), MLR (Monocyte-to-Lymphocyte Ratio), PLR (Platelet-to-Lymphocyte Ratio), SII (Systemic Immune-Inflammation Index: platelet count × neutrophil count/lymphocyte count), and SIRI (Systemic Inflammatory Response Index: neutrophil count x monocyte count/lymphocyte count) were calculated. Blood parameters were recorded at the following time points: within 24 h preoperatively (within one day preoperatively), within 24 h postoperatively (within the first postoperative day), within 24–72 h postoperatively (within the second or third postoperative days), within 72–120 h postoperatively (within the fourth or fifth postoperative days), and within 120–168 h postoperatively (within the sixth or seventh postoperative days). For the group that developed an abscess postoperatively, the following parameters were additionally recorded: postoperative day of abscess detection, radiological method of abscess detection, size of abscess detected radiologically, location of abscess, need for reoperation, and type of pathogen from reoperation.

### 2.3. Diagnostic Procedures

Complete blood counts of patients were measured from capillary or venous blood after a nurse or laboratory technician professionally collected the sample. Blood was analyzed on an XN-1000™ Automated Hematology Analyzer (Sysmex Corporation, Kobe, Japan) or an XS-800i™ Automated Hematology Analyzer (Sysmex Corporation, Kobe, Japan). CRP was analyzed from capillary or venous blood on a D7-CRP Hematology Analyzer (Dymind Biotechnology, Shenzhen, China), an AU400 or AU680 Clinical Chemistry Analyzer (Beckman Coulter, Brea, CA, USA).

If the first ultrasound finding was unclear, it was often repeated until the parameters of the appendix and periappendicular region were determined with greater certainty. All ultrasound examinations were performed on a Logiq E9^®^ device (GE Healthcare Systems, Chicago, IL, USA). If necessary, computed tomography was performed on a GE Revolution HD 128 (GE Healthcare Systems, Chicago, IL, USA).

### 2.4. Surgical Procedures

For an open appendectomy, the patient was placed in the supine position. A skin incision is made using the modified Lanz technique, typically a right lower quadrant incision, or, less commonly, a midline infraumbilical laparotomy, depending on surgeon preference and intraoperative considerations. Dissection is carried through the subcutaneous tissue and fascia, followed by entry into the peritoneal cavity. A swab is taken from the peritoneal cavity for microbiological analysis. The peritoneum is incised to expose the abdominal cavity, and gentle retraction is used to identify the cecum and the vermiform appendix. Once identified, the appendix is mobilized and exteriorized through the incision. The mesoappendix is carefully skeletonized from the tip toward the base or vice versa, utilizing absorbable sutures (Vicryl, Ethicon Inc., Somerville, NJ, USA). The appendiceal base is ligated with absorbable sutures, and the appendix is excised distal to the ligature. The mucosa at the appendiceal stump is cauterized or oversewn to prevent leakage. To further minimize postoperative stump-related complications, stump inversion is performed using a purse-string suture technique, ensuring secure inversion of the appendiceal mucosa into the cecum. The peritoneal cavity is thoroughly irrigated, and meticulous hemostasis is confirmed. The peritoneum, fascia, and skin are closed in layers using appropriate sutures.

For a laparoscopic appendectomy, the patient is positioned in the supine position with the operating table adjusted to facilitate optimal access. To enhance visualization and operative access, the patient is placed in a combined Trendelenburg position with left lateral tilt, depending on surgeon’s preference and intraoperative findings. After the Hasson or Veress needle technique, CO_2_ insufflation is initiated at a pressure of 8–10 mm Hg, adjusted according to the patient’s age, body habitus, and intra-abdominal compliance. Once adequate pneumoperitoneum is achieved, a 5 mm or 10 mm trocar is introduced through the same incision under direct visualization. Additional working trocars are placed under direct vision in the suprapubic and left lower quadrant regions, forming a three-trocar configuration with 5 mm and 10 mm ports, optimized for triangulation and instrument maneuverability. The cecum and appendix are identified. The mesoappendix is dissected using a harmonic scalpel (Ultracision, Ethicon Endo-Surgery, Cincinnati, OH, USA), or an ultrasonic surgical device (Sonicision, Medtronic, Minneapolis, MN, USA). The base of the appendix is secured with an endoloop (Vicryl Endoloop-0, Ethicon Endo-Surgery, Cincinnati, OH, USA) or polymeric ligating clips (e.g., Ligating Clips, Grena, Brentford, UK). The appendix is transected distal to the ligature. Care is taken to confirm secure ligation to prevent stump leakage. The resected appendix is placed into the specimen bag and extracted through the 10 mm trocar port, minimizing contamination of the wound. Extensive irrigation of the peritoneal cavity with warm, sterile saline is performed to reduce bacterial load and purulent material. The operative field is examined for hemostasis and residual contamination. The pneumoperitoneum is released, and trocars are removed. The fascial defects of ports are closed with absorbable sutures to prevent port-site herniation.

### 2.5. Outcome Measures

The primary outcome was to determine whether there were differences in preoperative and postoperative blood variables—such as blood count parameters, ratios, and inflammatory markers—between patients who developed intra-abdominal abscesses and those who did not, following surgery for perforated appendicitis. Additionally, the study aimed to assess whether the magnitude of percentage changes in blood variables across different perioperative time points showed differences between the groups. Secondary outcomes were to determine whether there were differences in clinical preoperative variables (duration of pain, nausea or vomiting, RLQ tenderness, rebound tenderness), radiological variables (appendix diameter, presence of collection, presence of fecalith), surgical variables (type of appendectomy, need for conversion, omentectomy, drainage, duration of surgery, time and season of appendectomy), length of hospital stay, length of stay in the intensive care unit between groups, as well as to determine the results of swabs taken and the types of antibiotics used in treatment. In the group that developed a postoperative intra-abdominal abscess, the time when the abscess was detected, the average size, and the location were determined.

### 2.6. Statistical Analysis

Descriptive statistics were used to characterize the patient cohort. Collected measurements were analyzed for normal distribution using the Shapiro–Wilk test. Categorical variables were expressed in absolute numbers and percentages. To assess differences in the distribution of categorical data, Fisher’s exact test or the chi-square test was used as appropriate. Continuous variables were analyzed using Student’s *t*-test or Mann–Whitney U test, as appropriate, based on data distribution. A multivariable logistic regression analysis was also performed on the preoperative variables of interest, with results expressed as adjusted odds ratios and 95% confidence intervals. The preoperative variables entered into this model were those that reached statistical significance in the univariate comparisons, namely rebound tenderness, leukocyte count, and neutrophil percentage, and the model was deliberately limited to these three predictors to respect the events per variable principle given the small number of abscess events. The discriminatory ability of the main preoperative and early postoperative biomarkers for later abscess development was additionally assessed with receiver operating characteristic analysis, and the area under the curve was reported together with the sensitivity and the specificity at the cut-off that maximized the Youden index. The data obtained were analyzed using the software program Microsoft Excel^®^ for Windows (Microsoft Corporation, Redmond, WA, USA) with the XLSTAT® add-in, version 2020.5.1 (Addinsoft, Paris, France). A significance level of 0.05 was used.

## 3. Results

During the observed ten-year period at the Children’s Hospital Zagreb, a total of 2332 children underwent appendectomy, either open or laparoscopically. Of these, 352 (15.1%) had perforated appendicitis. After applying the exclusion criteria, 206 patients remained for analysis. Of these, 176 (85.4%) patients did not develop an intra-abdominal abscess postoperatively, while 30 (14.6%) did (Figure 1).

The median age of all patients was 10.5 years (IQR 6.9–13.1). The youngest patient was 7 months old, and the oldest was 17.4 years old. There were 113 boys and 93 girls. An increased prevalence of comorbidities was observed in the group that developed an intra-abdominal abscess. Almost all of the children had not previously had abdominal surgery. The average duration of abdominal pain before presenting to the emergency department was 48.9 ± 32.2 h. The shortest duration of abdominal pain recorded in the anamnesis was only 2 h, and the longest was as long as 168 h, or one week. Nausea or vomiting was present in 77.2% of patients. Right lower quadrant tenderness was present in 87.4% of patients, while rebound tenderness was present in 38.3% of patients, being more in the group that developed an abscess postoperatively. The mean body temperature upon arrival at the emergency department was 38.1 ± 1.1 °C. Patient demographics and preoperative data between groups are shown in Table 1.

Two-thirds of patients underwent preoperative ultrasound. The mean diameter of the appendix was 9.7 ± 3.0 mm, with a larger diameter in the group that did not develop an abscess postoperatively. A collection on ultrasound was observed and described in 62 (45.6%) patients. Fecalith was observed and described in 31 (22.8%) patients. Patient radiological data between groups are shown in Table 2.

Almost all patients (96.6%) were surgically treated with the open method. Of the seven patients who underwent laparoscopic surgery, two were converted to open surgery in the group of patients who did not develop a postoperative abscess. Omentectomy was performed in 77 (37.4%) patients. Almost all patients (97.6%) were placed with a drain. The mean surgery duration was 99.9 ± 33.5 min. The highest number of perforated appendicitis occurred in the winter months (29.1%), and the surgery was most often performed between noon and 6 p.m. (38.8%). Surgical data of patients between groups are shown in Table 3.

Regarding the swabs’ results, the following bacteria were found in the group that did not develop an abscess postoperatively: *Bacteroides ovatus*, *Bacteroides distasonis*, *Bacteroides fragilis*, *Bifidobacterium* spp., *Clostridium perfringens*, *Clostridium ramosum*, *Enterobacter aerogenes*, *Enterobacter agglomerans*, *Enterococcus faecalis*, *Enterococcus faecium*, *Escherichia coli*, *Eubacterium lentum*, *Fusobacterium nucleatum*, *Haemophilus parainfluenzae*, *Klebsiella aerogenes*, *Klebsiella pneumoniae*, *Lactobacillus* spp., *Peptostreptococcus* sp., *Porphyromonas asaccharolytica*, *Prevotella melaninogenica*, *Pseudomonas aeruginosa*, *Salmonella enterica*, *Streptococcus constellatus*, *Streptococcus intermedius*, *Streptococcus viridans*, *Veillonella parvula*. The following bacteria dominated in terms of abundance: *Escherichia coli*, *Escherichia coli + Streptococcus intermedius*, *Escherichia coli + Pseudomonas aeruginosa*, *Escherichia coli + Bacteroides* spp. The following bacteria were found in the group that developed an abscess: *Bacteroides ovatus*, *Bacteroides fragilis*, *Clostridium* spp., *Enterococcus faecalis*, *Enterococcus faecium*, *Escherichia coli*, *Eubacterium* sp., *Fusobacterium* sp., *Gemella morbillorum*, *Klebsiella pneumoniae*, *Lactobacillus* spp., *Peptostreptococcus* sp., *Prevotella melaninogenica*, *Pseudomonas aeruginosa*, *Sphingomonas paucimobilis*, *Staphylococcus epidermidis*, *Streptococcus constellatus*, *Streptococcus intermedius*. The following bacteria dominated in terms of abundance: *Escherichia coli + Streptococcus intermedius*, *Escherichia coli + Pseudomonas aeruginosa*, *Escherichia coli + Bacteroides* spp. In the swabs after the necessary reoperation to remove the abscess, they dominated *Escherichia coli* and *Bacteroides* spp.

Regarding antibiotic therapy, the following combinations dominated in both groups: *ceftriaxone + metronidazole*, *cefuroxime + metronidazole*, *clindamycin + gentamicin*, *metronidazole + gentamicin*. Preoperatively, 154/176 (87.5%) patients in the group that did not develop an abscess received antibiotics, while 23/30 (76.7%) in the group that developed an abscess received them (*p* = 0.115). Accordingly, 22 of 176 children (12.5%) in the group without an abscess and 7 of 30 children (23.3%) in the group that developed one did not receive antibiotics in the preoperative period. In the group that did not develop a postoperative intra-abdominal abscess, antibiotic therapy was converted in 26/176 (14.8%) patients, while in the group that developed an abscess, therapy was converted in 23/30 (76.7%) patients (*p* < 0.001), most often immediately after ultrasound/CT confirmation of abscess presence and/or receipt of antibiogram, typically within the same clinical day. The most common targeted antibiotic adjustment involved switching to *piperacillin*/*tazobactam*.

As for the group that developed an intra-abdominal abscess postoperatively, the mean of abscess detection was 6.1 ± 1.8 days. All abscesses were detected by ultrasound, except one, which was detected by computed tomography. The mean size of the abscess was 35 ± 19.6 mm (min = 7 mm, max = 100 mm). Four patients had 2 or more abscesses verified. Reoperation was indicated in six patients to surgically remove the abscesses, while the others regressed on antibiotic therapy. The locations of abscesses are shown in Figure 2.

The mean total length of hospital stay for the group that did not develop an abscess was 10.3 ± 2.7 days, while for the group that developed an abscess, it was 16.9 ± 5.1 days (*p* < 0.001). In our cohort, most children undergoing surgery for perforated appendicitis were observed in the intensive care unit, primarily for close monitoring, with 169 of 206 patients (82%) spending at least 12 h in the intensive care unit after surgery. The length of stay in the intensive care unit was 2.7 ± 1.6 days for the first group and 4 ± 1.6 days for the second group (*p* < 0.001).

The mean values (±SD) of all blood count variables by groups, along with the corresponding *p*-values, are shown in Table 4.

Between the observed groups, leukocytes and neutrophils show a significant difference at all time points (↗—group 2), while lymphocyte percentage shows a significant difference within 24 h preoperatively and from day 4 onwards (↗—group 1). Absolute monocyte count shows a significant difference from day 2 onwards (↗—group 2). CRP shows a significant difference from day 4 onwards (↗—group 2). These are separate univariate comparisons at multiple postoperative time points and were not corrected for multiple testing, so they should be regarded as exploratory and the individual *p*-values should not be interpreted as confirmatory.

The mean values (±SD) of the ratios and indexes by group, along with the corresponding *p*-values, are shown in Table 5.

Observing the ratios and indexes after 72 h, except for PLR, all show a significant difference (↗—group 2). NLR and SII show a significant difference already within 24 h postoperatively, while SIRI shows a difference already after 24 h (↗—group 2). As with the other serial comparisons, these time-point differences are exploratory and hypothesis generating rather than confirmatory.

The average of individual percentage changes by groups, along with the corresponding *p*-values, are shown in Table 6.

NLR and SIRI in group 2 show a significant mean percentage increase between samples taken on the second and third days compared to samples taken on the fourth and fifth days. CRP in the specified interval shows an insignificant mean percentage decrease in group 2, while between samples taken on the 4th and 5th days compared to samples taken on the 6th and 7th days, it shows a significant slower mean percentage decrease.

Based on prior analyses, we included preoperatively determined rebound tenderness, leukocyte count, and neutrophil percentage in the multivariable logistic regression model (Table 7).

We assessed multicollinearity between WBC count and neutrophil percentage by calculating the Pearson correlation coefficient. The correlation was 0.69, below the commonly accepted threshold (r < 0.8), indicating that multicollinearity was not a significant concern. Among the variables examined, preoperative neutrophil percentage was the only independent predictor of abscess development (adjusted OR 1.10, 95% CI 1.01 to 1.19, *p* = 0.034), with higher values associated with increased odds of abscess formation. Given the limited number of abscess events, the model was restricted to three preoperative predictors in line with the events-per-variable principle. In receiver operating characteristic analysis the preoperative neutrophil percentage showed only modest discrimination for later abscess development, with an area under the curve of 0.68, and at a cut-off of 86.6% the sensitivity was 65% and the specificity was 68%. The preoperative leukocyte count and C-reactive protein showed similarly modest discrimination, with areas under the curve of 0.64 and 0.65, while among the early postoperative measurements the leukocyte count on the second and third postoperative days provided the best single discrimination, with an area under the curve of 0.74. Taken together, these values indicate that no individual biomarker reached an accuracy that would justify its use as a stand-alone predictive test, which is consistent with our interpretation of the biomarkers as an adjunct to clinical assessment and imaging rather than as a replacement for them.

## 4. Discussion

This retrospective study is, to our knowledge, one of the first to investigate laboratory biomarkers for the early signaling of the development of postoperative intra-abdominal abscesses in a pediatric population after surgical treatment of perforated appendicitis. Analyzing data from 206 children, over a ten-year period, we found that certain blood biomarkers showed significant changes within 72 h after surgery in patients who developed abscesses compared to those who did not. Notably, these biomarkers may signal an increased likelihood of abscess detection earlier than imaging is usually performed in current clinical practice, as radiological detection typically occurs around the sixth day after surgery. This early postoperative signal should be distinguished from later measurements obtained around or after the time of radiological diagnosis, which reflect the evolving clinical course rather than a purely predictive signal. The findings highlight the potential of blood biomarkers to improve the timely postoperative management of abscesses after perforated appendicitis in children, and may complement, rather than replace, clinical assessment and imaging in the postoperative care.

In a four-year study by Fike et al. [15], as many as 50.8% of patients developed a postoperative abscess after laparoscopic appendectomy for perforated appendicitis. In contrast to our study, in their study, almost all abscesses were detected by CT, which exposed the children to additional ionizing radiation. Similarly to our results, abscesses in hospitalized patients were detected radiologically after one week (7 ± 1.7 days). In contrast to their results, which did not show a difference in the initial leukocyte count, our results suggest that there is a significant difference in the leukocyte count within the first seven days after surgery. They also found that patients with developed abscesses were older with a higher body mass index. Similar to our results, theirs indicated that the length of hospital stay in the group with developed abscesses increased by one week. Most abscesses were treated with drainage, aspiration, and prolonged outpatient antibiotic therapy, with a high success rate. In our study, all patients who developed a postoperative abscess received a drain during the initial surgery, so antibiotic therapy was sufficient for most patients (only six required reoperation).

According to the study by Gorter et al. [16], most postoperative abscesses were located in the right lower quadrant, as in our study. The authors emphasized that noninvasive management, mainly for patients with small or uncomplicated abscesses and good clinical conditions, can be effective and has fewer complications and hospital days. They also noted that treatment choice depends on factors like abscess size, location, and patient stability. Overall, their findings support noninvasive strategies as a safe and effective option for managing postoperative intra-abdominal abscess in children with stable clinical conditions [16]. The study by Emil et al. [17] aimed to identify risk factors for postoperative abscess formation following perforated appendicitis and to evaluate criteria for performing percutaneous drainage. Analysis of data from children treated over five years, the researchers found that 14.8% developed abscesses post-surgery, which is almost identical to the number in our study. Using logistic regression analysis, key factors linked to abscess development included elevated white blood cell counts, presence of bowel obstruction at presentation, diffuse peritonitis with a dominant abscess at surgery, and the involvement of a particular surgeon. Conversely, the presence of fever or significant pain at the time of abscess diagnosis was associated with the decision to perform drainage [17]. According to Obinwa et al., of all the clinical variables evaluated, preoperative temperature ≥ 37.5 °C was most discriminatory for advanced appendicitis. Significant other discriminatory clinical variables were WBC count ≥ 15,100 /µL, preoperative anorexia, and rebound tenderness [18]. Although our study observed a higher prevalence of comorbidities among patients who developed intra-abdominal abscesses compared to those who did not, this difference did not reach statistical significance. This trend suggests that comorbidities may influence abscess formation, but larger studies are necessary to clarify their potential impact on postoperative outcomes in pediatric perforated appendicitis.

Our study focused on perforated appendicitis, which was mostly detected preoperatively on imaging, so most surgeons chose an open approach. Considering all types of appendicitis, open appendectomy has always been associated with higher incidence rates of surgical site infections and sepsis compared to laparoscopic procedures [19,20].

Although the practice at our hospital is for all patients to receive a drain after surgery for perforated appendicitis, recent high-quality evidence challenges this long-held belief. A 2025 Cochrane review concluded that drainage did not significantly reduce intra-abdominal abscesses, wound infection rates, or overall complications, but instead may prolong hospital stay due to a higher incidence of superficial wound infections, fistulas, bowel obstructions, and ileus [21]. International guidelines have gradually moved away from supporting routine intra-abdominal drainage, especially in the context of minimally invasive surgery. They advocate a consensus towards individualized, selective use of drains rather than prophylactic placement [19].

Although the practice in our hospital is to routinely take swabs for perforated appendicitis, based on which a significantly higher percentage of antibiotic therapy was converted in the group that developed an abscess (76.7% vs. 14.8%), certain studies advocate the view that swabs have little clinical value, that is, that this practice does not reduce postoperative morbidity [22,23]. The WSES guidelines are against the routine use of intraoperative cultures and recommend their use in case of nosocomial infections, immunocompromised patients, or in cases with known exposure to resistant organisms [19]. It is also important to note that copious peritoneal lavage with saline is standard practice in our hospital, especially in the perforated appendicitis group. Recent studies suggest that aspiration alone is equivalent to irrigation in preventing intra-abdominal abscesses and even better in terms of operative time and reoperation rate [24,25]. It has also been argued that the use of antiseptic irrigation solutions is more important than the mechanical act of saline irrigation [26,27]. A minority of children in both groups did not receive antibiotics in the preoperative period, and this proportion did not differ significantly between those who did and did not develop an abscess (*p* = 0.115), so preoperative antibiotic administration is unlikely to have been a major determinant of postoperative abscess formation in our cohort.

As in our study, in cases where the postoperative abscess is small, well localized and not associated with signs of sepsis or generalized peritonitis, antibiotic therapy is the basis of conservative treatment and is a safe alternative to percutaneous or surgical drainage [28]. Although in our study most of the therapy was converted to a piperacillin-tazobactam combination, ceftriaxone in combination with metronidazole or carbapenems are also options [29]. Switching to oral therapy is appropriate when the patient is afebrile, tolerates oral intake, and shows clinical improvement, whereas failure to respond should prompt reconsideration of source control measures, including image-guided drainage [30,31]. Svetanoff et al. found that patients with abscess sizes < 20 cm^2^ who were not drained had no difference in clinical outcomes compared to patients with abscess sizes > 20 cm^2^ who were drained [32]. In cases where conservative treatment fails either due to inadequate response to antibiotic therapy or unsuccessful drainage, surgical reintervention becomes necessary [4,33]. It is important to note that caution should be exercised when interpreting most of the previous conclusions from other studies, as most studies were conducted in adult patients, and applicability to the pediatric population may be limited.

Regarding biomarkers, according to the study by Son et al. and Mao et al. [2,34], elevated CRP at admission can predict the development of postoperative intra-abdominal abscess formation, while according to the study by Zhong et al., the occurrence of post-operative organ/space infection was associated with a lower level of pre-operative lymphocyte-C-reactive protein (OR 14.42, *p*  <  0.001) [35]. According to the study by Obayashi et al. [36], preoperative WBC and CRP indicated an increased risk of developing wound infections and intra-abdominal abscesses. If WBC (/μL) > 16,500, CRP > 3.1 mg/dL, and appendix maximum short diameter on diagnostic imaging > 11.4 mm, complications increased sixfold. According to Serradilla et al., the occurrence of intra-abdominal abscess was significantly associated with preoperative hyponatremia, elevated CRP, appendix perforation, and wound infection, but the multivariate analysis dismissed the value of the CRP as an abscess predictor [37]. In our study, there was no significant difference in CRP values between groups at the time of admission, although the mean value was higher in the group that developed it. According to the study by Moreno-Alfonso et al. NLR, PLR, CRP, and PCT values at the time of admission were higher in the group that developed a postoperative abscess (*p* < 0.001), but it is important to note that in their study, all types of acute appendicitis were included, not only perforated appendicitis as in ours [38]. According to the study by Delgado-Miguel et al., NLR presented an area under the curve (AUC) of 0.85, significantly higher than the determination of leukocytes (AUC 0.69, *p* < 0.001), neutrophils (AUC 0.74, *p* < 0.001), fibrinogen (AUC 0.68, *p* < 0.001), and C-reactive protein (AUC 0.73, *p* < 0.001). In this study, all types of appendicitis were included, and the findings were sampled at the time of admission to the hospital [39]. In contrast to blood sampling at the time of admission, Zheng et al. [40] evaluated the utility of postoperative laboratory tests in predicting the development of intra-abdominal abscess. The results showed that there was a large heterogeneity and overlap in postoperative laboratory values between patients who developed intra-abdominal abscess and those who did not. While patients who developed intra-abdominal abscess had a higher postoperative median leukocyte count (10.8 vs. 8.4, *p* = 0.003) and a smaller decrease in leukocyte count (−4.9 vs. −7.4, *p* = 0.01), no cutoff value for any of the laboratory values tested specifically predicted abscess formation. Postoperative median absolute neutrophil count (7.4 vs. 4.0, *p* = 0.15), platelet count (360 vs. 353, *p* = 0.98), and C-reactive protein (8.20 vs. 5.32, *p* = 0.06) were not significantly different [40]. According to the study by Lodwick et al., patients with intra-abdominal abscess had higher rates of leukocytosis (44.3% vs. 12.3%, *p* < 0.001) and higher rates of age-based lymphopenia (84% vs. 46%, *p* < 0.001). Postoperative intra-abdominal abscess was independently associated with leukocytosis (OR 3.65, *p* < 0.001) and lymphopenia (OR 4.46, *p* < 0.001) when the interval between admission and abscess detection, or discharge in patients who did not develop an abscess, was considered [41]. As in our study, and in the study by Dickinson et al., late persistent leukocytosis from days 5 to 7 was associated with the presence of an abscess [42]. However, it is important to note that, despite these promising findings, biomarkers alone have limited specificity in definitively diagnosing postoperative abscesses. As alluded to in various studies, imaging remains the gold standard for confirmation [7,43]. While biomarkers may signal an increased likelihood or early inflammatory response, they do not replace the need for radiological studies, which can definitively identify abscess formation. It is also important to recognize that persistent elevation or increase in inflammatory biomarkers such as leukocytes, neutrophils, CRP, SII, and SIRI postoperatively may reflect ongoing inflammatory processes rather than definitively indicating abscess formation. These findings highlight that biomarkers alone are limited in specificity and should be interpreted within the broader clinical context, including physical examination and imaging studies. Therefore, in clinical practice, biomarkers should be viewed as adjuncts to, rather than substitutes for, imaging techniques. It is also important to acknowledge that this study primarily focuses on the early postoperative period, during which biomarker changes may serve as useful signals of intra-abdominal abscess formation. However, late or delayed abscess formation, although rare, can still occur. Therefore, while our findings highlight the potential of blood biomarkers for early signaling, further research with extended follow-up is necessary to evaluate their utility in predicting late abscess development and to establish comprehensive postoperative monitoring protocols.

An unexpected observation in our cohort was that the mean sonographic appendix diameter was smaller in children who developed an abscess (8.1 ± 2.3 mm) than in those who did not (10.0 ± 3.0 mm, *p* = 0.015). This finding should be interpreted with caution, because ultrasound was performed and the appendix diameter measured in only a subset of patients, and the number of measured children in the abscess group was small. A smaller measured diameter may reflect a decompressed appendix after perforation, a contained or walled-off process that is sonographically less conspicuous, or the well-recognized operator dependence and limited sensitivity of ultrasound in the perforated setting. Rather than indicating milder disease, a smaller or poorly visualized appendix on ultrasound may therefore accompany perforation that later evolves into an abscess, and this observation warrants prospective evaluation.

The concept of early detection in our study should be understood as an earlier biochemical signal rather than a proven earlier clinical diagnosis. The significant differences in leukocytes, neutrophils, and the derived inflammatory indices were already present within the first 72 h after surgery, whereas the abscess was detected radiologically at a mean of 6.1 days, and 29 of 30 abscesses were diagnosed on the fourth postoperative day or later. The biomarker changes within the first 72 h therefore preceded the radiological diagnosis in almost all cases and can be regarded as an early biochemical signal and adjunctive risk information rather than a validated predictive test, while values obtained on the fourth to seventh postoperative days increasingly overlapped with or followed the moment of diagnosis and any escalation of treatment and should be interpreted as reflecting the evolving clinical course rather than a purely predictive signal. These biomarker trends are not diagnostic on their own and are most informative when they are integrated with the clinical picture, including persistent or recurrent fever, abdominal findings such as rebound tenderness, feeding intolerance, and the overall postoperative trajectory, which together should prompt targeted imaging.

Despite the comprehensiveness of the study, there are some limitations. First and foremost, the retrospective nature of the study and the data collected from a single tertiary center may limit the generalizability of the findings to different populations, healthcare facilities, and geographic regions. Variations in surgical techniques and postoperative treatment, including antibiotic regimens, could influence the outcomes. Although 206 patients with perforated appendicitis were analyzed, only 14.6% developed intra-abdominal abscesses. This relatively small number of events may reduce the statistical power to detect subtle differences. Moreover, the relatively wide intervals between measurement time points, particularly the two-day gaps, could have influenced the detection of dynamic biomarker changes, potentially leading to delayed recognition of early inflammatory responses. Additionally, the interval from hospital presentation to surgical intervention was not specifically analyzed, which may act as a confounding factor influencing inflammatory markers and postoperative outcomes. Due to the limited number of intra-abdominal abscess cases relative to the total sample size, a multivariable logistic regression analysis was performed using only three variables of interest. External factors such as nutritional status or genetic predispositions were not taken into account, which could influence inflammatory responses and biomarker levels. Over the 10-year period, surgical treatment was predominantly performed via open appendectomy, which contrasts with the current trend toward minimally invasive laparoscopic approaches. This may influence postoperative complication rates and limits applicability to centers utilizing predominantly laparoscopic techniques. Additionally, routine intra-abdominal drain placement was performed in all cases that developed a postoperative intra-abdominal abscess, despite recent guidelines recommending a more selective approach, which may have affected postoperative outcomes, including abscess formation. Also, surgical treatment was performed by several different surgeons, as were ultrasounds performed by several different radiologists. Despite the excellence of our radiologists, it is important to note that ultrasound alone has limited reliability in definitively diagnosing abscess formation, as fluid collections may appear sonolucent and nonspecific. Additionally, it is possible that some small intra-abdominal abscesses remained undiagnosed or were not detected, which may have resulted in an underestimation of the true incidence. Several additional limitations should be acknowledged. The timing of postoperative clinical suspicion, fever, abdominal symptoms, and the imaging request was not systematically recorded in relation to the biomarker sampling, so we could not directly compare the moment of biochemical change with the moment of clinical or radiological suspicion. Laboratory values obtained on the later postoperative days may in some abscess cases have been drawn around or after the radiological diagnosis and after escalation of antibiotic therapy, which limits their interpretation as purely predictive markers. The intraoperative severity of intra-abdominal disease was not graded in a standardized way, and although the presence of a fecalith and of a sonographic collection was recorded, the extent of peritonitis as localized or diffuse was not available for retrospective analysis. In addition, the postoperative drainage protocol could not be characterized in detail, as only the presence of a drain was documented, whereas the exact day of drain removal and the total duration of drainage were not recorded, so a reliable median or mean drain duration could not be reported for this cohort. Finally, a standardized duration of structured follow-up after discharge was not defined, and although children requiring further care returned to the same tertiary center, delayed abscesses that presented elsewhere or remained asymptomatic could have been missed. A further limitation concerns the statistical treatment of the serial laboratory data. The postoperative values are repeated measurements obtained within the same patients and are therefore not statistically independent, and the biomarkers were compared across several postoperative time points with separate univariate tests. These time-point comparisons should be regarded as exploratory and hypothesis generating, because we did not apply a formal correction for multiple comparisons or a longitudinal mixed-effects model, so the possibility of type I error is increased and the individual *p*-values should be interpreted with corresponding caution. In addition, as in any retrospective cohort, children with a more difficult postoperative course were more likely to undergo repeated blood sampling and imaging, which can create differential ascertainment between those later diagnosed with an abscess and those who recovered uneventfully. Modification of antibiotic therapy was also more frequent in the abscess group and often followed radiological confirmation, so that some of the later laboratory values reflect both the complication and its treatment rather than a purely spontaneous course. Finally, because almost all children in this cohort were treated with open appendectomy and almost all received an intra-abdominal drain, the study population differs substantially from many contemporary pediatric surgical centers where laparoscopy without routine drainage predominates, and the external validity of our findings should therefore be interpreted with particular caution.

## 5. Conclusions

This study shows that certain biomarkers in the blood, such as leukocytes, neutrophils, lymphocytes, monocytes, and their specific ratios and indices, show significant differences and changes within the early postoperative period in children who develop intra-abdominal abscesses after perforated appendicitis compared to those who do not develop them. These findings suggest that routine blood tests at defined intervals may serve as timely indicators for early risk stratification, and, when interpreted together with clinical findings and confirmatory imaging, may support earlier suspicion of abscess formation and more timely imaging, although on their own they cannot establish the diagnosis or determine the need for intervention. Blood biomarkers should be regarded as an adjunct that raises or lowers the suspicion of abscess formation rather than as a stand-alone diagnostic test, since imaging remains necessary for definitive diagnosis. Because the present findings represent associations observed in a single center and were not externally validated, they should be confirmed in prospective multicenter studies before routine laboratory monitoring can be recommended to guide earlier intervention.

## Figures and Tables

**Figure 1:**
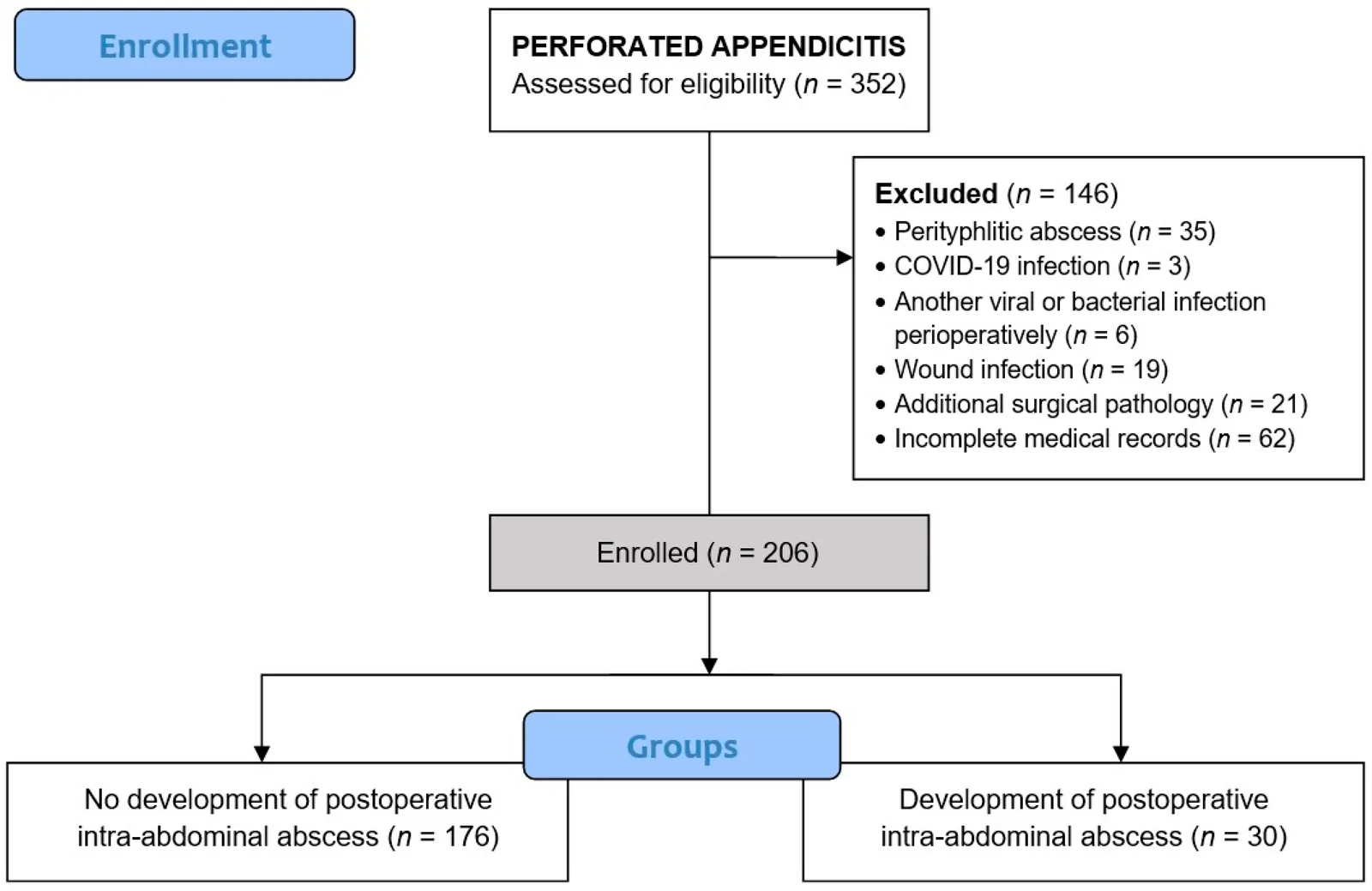
Flowchart of the study.

**Figure 2:**
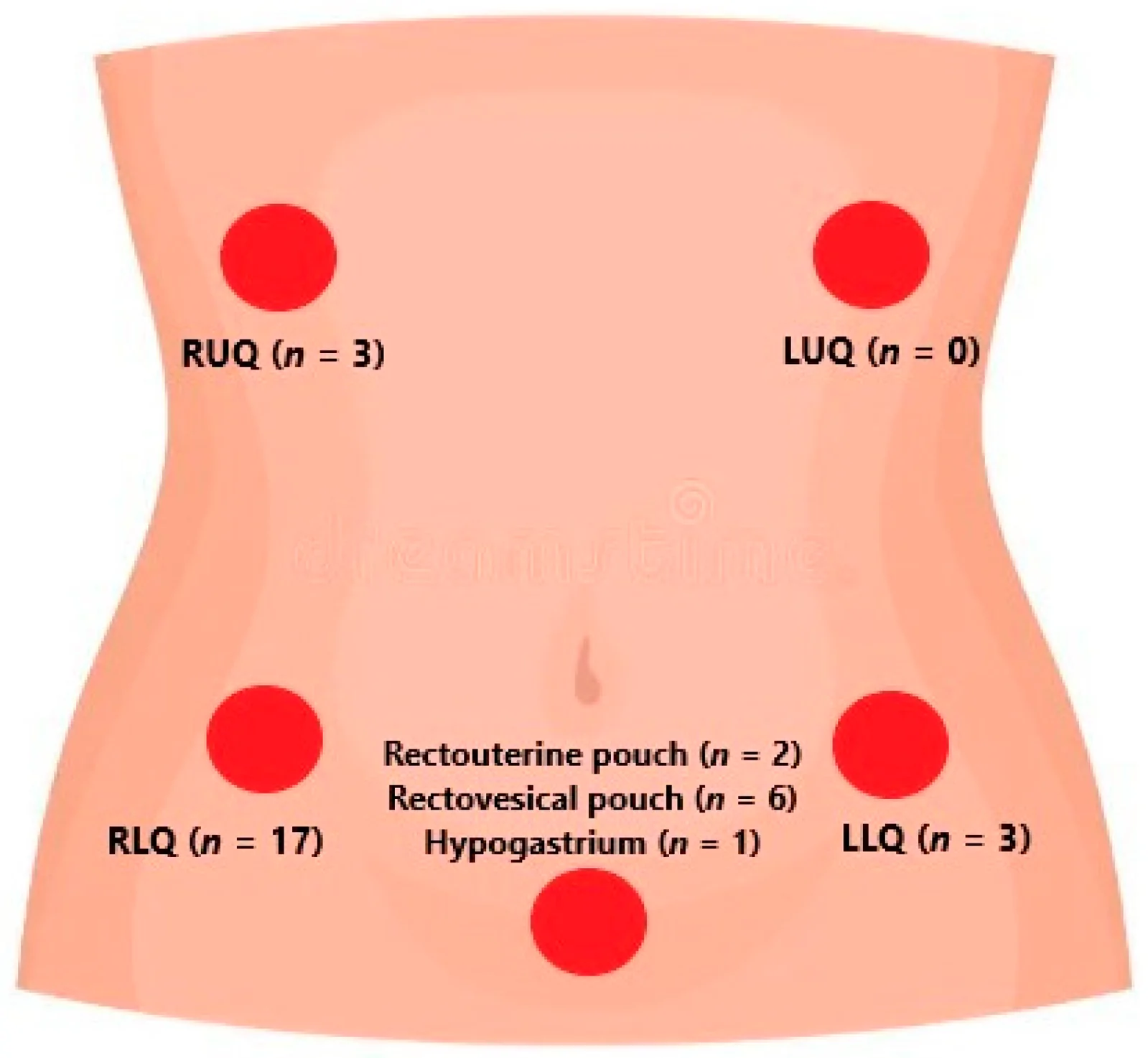
Locations of postoperative abscesses (RUQ—right upper quadrant, LUQ—left upper quadrant, RLQ—right lower quadrant, LLQ—left lower quadrant).

**Table 1:** Demographic and preoperative data of patients (*n* = 206).

Variables	Group Without Abscess Development (*n* = 176)	Group with Abscess Development (*n* = 30)	*p*
Age (months)			0.066 *
mean ± SD	116.6 ± 48.9	134.7 ± 53.1	
min–max	7–209	22–205	
Sex			
male, female	95, 81	18, 12	0.540 ^‡^
Comorbidity, *n* (%)			
Yes	25 (14.2)	8 (26.7)	0.085 ^‡^
No	151 (85.8)	22 (73.3)	
Previous abdominal surgery, *n* (%)			
Yes	3 (1.7)	0 (0)	1.000 ^†^
No	173 (98.3)	30 (100)	
Duration of abdominal pain (hours)	48.9 ± 33.4	48.6 ± 26.6	0.959 *
Nausea or vomiting, *n* (%)			
Yes	135 (76.7)	24 (80)	0.691 ^‡^
No	41 (23.3)	6 (20)	
RLQ tenderness, *n* (%)			
Yes	156 (88.6)	24 (80)	0.188 ^‡^
No	20 (11.4)	6 (20)	
Rebound tenderness, *n* (%)			
Yes	64 (36.4)	15 (50)	0.027 ^‡^
No	112 (63.6)	15 (50)	
Body temperature (°C)	38.2 ± 0.9	37.9 ± 1.9	0.199 *

SD—standard deviation, min—minimum, max—maximum, RLQ—right lower quadrant, * Student’s *t*-test, ^‡^ Chi-square test, ^†^ Fisher’s exact test.

**Table 2:** Preoperative radiological data of patients (*n* = 206).

Variables	Group Without Abscess Development (*n* = 176)	Group with Abscess Development (*n* = 30)	*p*
Preoperative US, *n* (%)			
Yes	115 (65.3)	21 (70)	0.618 ^‡^
No	61 (34.7)	9 (30)	
Appendix diameter (mm)	10.0 ± 3.0	8.1 ± 2.3	0.015 *
Collection, *n* (%)			
Yes	53 (46.1)	9 (42.9)	0.785 ^‡^
No	62 (53.9)	12 (57.1)	
Fecalith, *n* (%)			
Yes	29 (25.2)	2 (9.5)	0.159 ^†^
No	86 (74.8)	19 (90.5)	

US—ultrasound, mm—millimeter, * Student’s *t*-test, ^‡^ Chi-square test, ^†^ Fisher’s exact test.

**Table 3:** Surgical data on patients undergoing appendectomy (*n* = 206).

Variables	Group Without Abscess Development (*n* = 176)	Group with Abscess Development (*n* = 30)	*p*
Appendectomy, *n* (%)			
Open	170 (96.6)	29 (96.7)	1.000 ^†^
Laparoscopic	6 (3.4)	1 (3.3)	
Conversion, *n* (%)			
Yes	2 (33.3)	0 (0)	1.000 ^†^
No	4 (66.7)	1 (100)	
Omentectomy, *n* (%)			
Yes	65 (36.9)	12 (40)	0.748 ^‡^
No	111 (63.1)	18 (60)	
Drainage, *n* (%)			
Yes	171 (97.2)	30 (100)	1.000 ^†^
No	5 (2.8)	0 (0)	
Duration of surgery (min)	98.8 ± 34.2	104.1 ± 31.2	0.448 *
Season, *n* (%)			
Spring	43 (24.4)	8 (26.7)	0.475 ^‡^
Summer	46 (26.1)	4 (13.3)	
Autumn	38 (21.6)	7 (23.3)	
Winter	49 (27.9)	11 (36.7)	
Time of surgery, *n* (%)			
Midnight to 6:00 AM	19 (10.8)	2 (6.7)	0.241 ^‡^
6:00 AM to Noon	35 (19.9)	9 (30)	
Noon to 6:00 PM	66 (37.5)	14 (46.7)	
6:00 PM to Midnight	56 (31.8)	5 (16.6)	

min—minute, AM—ante meridiem, PM—post meridiem, * Student’s *t*-test, ^‡^ Chi-square test, ^†^ Fisher’s exact test.

**Table 4:** Blood count variables of patients (*n* = 206).

Variables	Within 24 h Preoperatively		Within 24 h Postoperatively		Within 24–72 h Postoperatively		Within 72–120 h Postoperatively		Within 120–168 h Postoperatively	
	G1	G2	G1	G2	G1	G2	G1	G2	G1	G2
Erythrocytes (×10^12^/L)	4.9 ± 0.5	4.9 ± 0.6	4.2 ± 0.4	4.2 ± 0.5	4.2 ± 0.4	4.1 ± 0.4	4.4 ± 0.5	4.4 ± 0.5	4.7 ± 0.5	4.5 ± 0.5
*p*	0.853 *		0.918 *		0.487 *		0.507 *		0.020 *	
Hemoglobin (g/L)	131.6 ± 14.4	132.8 ± 17.6	115.3 ± 12.6	114.3 ± 16.0	112.7 ± 12.4	111.2 ± 14.1	119.6 ± 15.6	117.3 ± 15.8	126.8 ± 14.7	120.7 ± 15.4
*p*	0.700 *		0.693 *		0.565 *		0.481 *		0.052 *	
Hematocrit (L/L)	0.4 ± 0.1	0.4 ± 0.1	0.4 ± 0.1	0.4 ± 0.1	0.4 ± 0.1	0.3 ± 0.1	0.4 ± 0.1	0.4 ± 0.1	0.4 ± 0.1	0.4 ± 0.1
*p*	0.861 *		0.761 *		0.618 *		0.582 *		0.060 *	
MCV (fL)	82.5 ± 5.2	82.4 ± 5.6	85.1 ± 4.7	84.6 ± 5.3	84.6 ± 5.7	84.8 ± 5.9	83.1 ± 5.1	83.3 ± 5.4	82.8 ± 4.6	83.3 ± 5.7
*p*	0.912 *		0.598 *		0.839 *		0.879 *		0.636 *	
MCH (pg)	27.2 ± 2.0	27.2 ± 2.1	27.3 ± 1.7	27.1 ± 2.0	27.1 ± 1.9	27.1 ± 1.9	27.0 ± 2.0	26.9 ± 1.9	26.8 ± 1.8	26.9 ± 1.9
*p*	0.967 *		0.549 *		0.981 *		0.823 *		0.871 *	
MCHC (g/L)	329.2 ± 11.8	329.9 ± 12.6	320.7 ± 10.2	319.9 ± 9.1	320.3 ± 10.3	319.6 ± 10.7	324.2 ± 12.3	322.7 ± 11.0	324.0 ± 11.8	323.1 ± 13.0
*p*	0.787 *		0.703 *		0.718 *		0.529 *		0.714 *	
RDW-CV (%)	13.2 ± 1.0	13.5 ± 1.3	13.0 ± 1.0	13.2 ± 1.3	12.9 ± 1.1	13.1 ± 1.4	12.8 ± 1.2	13.0 ± 1.3	12.8 ± 1.2	13.1 ± 1.4
*p*	0.248 *		0.390 *		0.337 *		0.484 *		0.338 *	
Platelets (×10^9^/L)	290.4 ± 76.0	276.2 ± 81.4	235.3 ± 64.8	249.6 ± 79.9	252.0 ± 72.5	265.5 ± 81.8	324.8 ± 99.1	356.3 ± 122.0	428.0 ± 112.9	473.5 ± 146.3
*p*	0.394 *		0.292 *		0.369 *		0.139 *		0.070 *	
MPV (fL)	9.6 ± 0.9	9.7 ± 0.9	9.7 ± 0.8	9.8 ± 1.0	9.6 ± 0.8	9.7 ± 0.8	9.5 ± 0.8	9.5 ± 0.8	9.4 ± 0.8	9.2 ± 0.7
*p*	0.569 *		0.523 *		0.659 *		0.929 *		0.126 *	
Leukocytes (×10^9^/L)	18.2 ± 6.6	21.0 ± 5.2	13.0 ± 4.8	15.2 ± 4.9	10.6 ± 3.9	14.1 ± 4.3	10.1 ± 3.5	15.0 ± 6.7	9.9 ± 3.7	14.7 ± 5.7
*p*	0.032 *		0.026 *		<0.001 *		<0.001 *		<0.001 *	
Neutrophils (%)	81.3 ± 9.8	86.1 ± 5.4	79.9 ± 8.3	83.8 ± 4.4	70.9 ± 11.1	76.0 ± 7.3	66.6 ± 8.7	73.7 ± 8.3	62.6 ± 10.6	71.0 ± 8.2
*p*	0.017 *		0.046 *		0.050 *		<0.001 *		<0.001 *	
(×10^9^/L)	15.0 ± 5.9	17.8 ± 4.6	10.5 ± 4.6	13.6 ± 4.7	7.7 ± 3.9	10.4 ± 3.1	6.7 ± 2.9	11.4 ± 6.3	6.4 ± 3.3	10.6 ± 5.2
*p*	0.021 *		0.006 *		0.004 *		<0.001 *		<0.001 *	
Lymphocytes (%)	10.0 ± 6.6	7.0 ± 3.5	11.1 ± 6.3	8.3 ± 3.4	17.4 ± 9.0	13.5 ± 6.4	20.9 ± 8.5	14.8 ± 5.9	25.5 ± 9.8	17.4 ± 7.1
*p*	0.029 *		0.055 *		0.067 *		0.001 *		<0.001 *	
(×10^9^/L)	1.7 ± 1.1	1.4 ± 0.8	1.3 ± 0.7	1.3 ± 0.4	1.7 ± 0.9	1.8 ± 0.9	2.0 ± 1.1	2.0 ± 0.8	2.3 ± 0.9	2.2 ± 0.6
*p*	0.283 *		0.749 *		0.644 *		0.917 *		0.635 *	
Monocytes (%)	7.7 ± 3.1	6.6 ± 2.4	8.3 ± 3.1	7.5 ± 1.6	9.5 ± 2.7	8.9 ± 1.5	9.7 ± 2.3	9.4 ± 2.9	8.8 ± 1.9	8.8 ± 2.3
*p*	0.088 *		0.264 *		0.382 *		0.564 *		0.959 *	
(×10^9^/L)	1.4 ± 0.6	1.3 ± 0.6	1.0 ± 0.5	1.2 ± 0.4	1.0 ± 0.4	1.2 ± 0.3	0.9 ± 0.4	1.3 ± 0.5	0.9 ± 0.4	1.2 ± 0.5
*p*	0.855 *		0.228 *		0.008 *		<0.001 *		<0.001 *	
Eosinophils (%)	0.2 ± 0.3	0.2 ± 0.3	0.3 ± 0.7	0.3 ± 0.5	1.8 ± 1.7	1.3 ± 1.2	2.8 ± 2.3	1.8 ± 1.2	2.7 ± 2.2	2.3 ± 1.6
*p*	0.374 *		0.580 *		0.137 *		0.028 *		0.249 *	
(×10^9^/L)	0.1 ± 0.1	0.1 ± 0.1	0.1 ± 0.1	0.1 ± 0.1	0.2 ± 0.2	0.2 ± 0.2	0.2 ± 0.2	0.2 ± 0.1	0.2 ± 0.2	0.3 ± 0.2
*p*	0.848 *		0.980 *		0.922 *		0.766 *		0.434 *	
Basophils (%)	0.2 ± 0.1	0.3 ± 0.1	0.3 ± 0.2	0.2 ± 0.1	0.3 ± 0.2	0.3 ± 0.1	0.5 ± 0.2	0.4 ± 0.2	0.6 ± 0.3	0.6 ± 0.4
*p*	0.288 *		0.412 *		0.082 *		0.210 *		0.436 *	
(×10^9^/L)	0.1 ± 0.1	0.1 ± 0.1	0.1 ± 0.1	0.1 ± 0.1	0.1 ± 0.1	0.1 ± 0.1	0.1 ± 0.1	0.1 ± 0.1	0.1 ± 0.1	0.1 ± 0.1
*p*	0.015 *		0.820 *		0.341 *		0.008 *		0.002 *	
IG (%)	0.5 ± 0.4	0.5 ± 0.3	0.5 ± 0.4	0.5 ± 0.2	0.8 ± 1.1	0.7 ± 0.8	1.1 ± 1.5	1.6 ± 1.3	1.1 ± 1.0	2.1 ± 1.7
*p*	0.916 *		0.980 *		0.729 *		0.132 *		<0.001 *	
(×10^9^/L)	0.1 ± 0.1	0.1 ± 0.1	0.1 ± 0.1	0.1 ± 0.1	0.1 ± 0.2	0.1 ± 0.1	0.1 ± 0.2	0.3 ± 0.3	0.1 ± 0.2	0.3 ± 0.3
*p*	0.785 *		0.539 *		0.930 *		0.021 *		<0.001 *	
CRP (mg/L)	114.0 ± 82.8	147.4 ± 72.6	178.3 ± 72.5	214.2 ± 49.5	146.5 ± 82.8	174.1 ± 75.1	89.8 ± 68.1	148.3 ± 67.9	30.1 ± 35.9	84.0 ± 53.1
*p*	0.073 *		0.061 *		0.135 *		<0.001 *		<0.001 *	

G1—group without abscess development (*n* = 176), G2—group with an abscess development (*n* = 30), MCV—mean corpuscular volume, MCH—mean corpuscular hemoglobin, MCHC—mean corpuscular hemoglobin concentration, RDW-CV—red cell distribution width—coefficient of variation, MPV—mean platelet volume, g—gram, L—liter, fL—femtoliter, pg—picogram, IG—immature granulocytes, CRP—C-reactive protein, mg—milligram. * Student’s *t*-test.

**Table 5:** Ratios and indexes of patients (*n* = 206).

Variables	Within 24 h Preoperatively		Within 24 h Postoperatively		Within 24–72 h Postoperatively		Within 72–120 h Postoperatively		Within 120–168 h Postoperatively	
	G1	G2	G1	G2	G1	G2	G1	G2	G1	G2
NLR	12.1 ± 8.8	15.3 ± 7.1	9.5 ± 5.1	12.3 ± 6.7	6.0 ± 5.2	7.2 ± 4.1	3.8 ± 1.9	6.2 ± 3.7	3.1 ± 2.0	4.9 ± 2.5
*p*	0.088 *		0.033 *		0.304 *		<0.001 *		<0.001 *	
NMR	12.3 ± 5.4	15.0 ± 5.7	11.6 ± 6.8	11.9 ± 3.6	8.7 ± 4.9	8.8 ± 1.9	7.4 ± 2.6	8.9 ± 4.0	7.5 ± 2.3	8.8 ± 3.0
*p*	0.024 *		0.811 *		0.940 *		0.017 *		0.020 *	
MLR	1.0 ± 0.6	1.1 ± 0.4	0.9 ± 0.5	1.0 ± 0.4	0.7 ± 0.4	0.8 ± 0.4	0.5 ± 0.2	0.7 ± 0.3	0.4 ± 0.2	0.6 ± 0.3
*p*	0.722 *		0.232 *		0.145 *		0.002 *		<0.001 *	
PLR	231.0 ± 140.3	234.5 ± 111.4	211.5 ± 94.2	229.6 ± 87.5	185.8 ± 95.3	178.0 ± 97.2	191.1 ± 75.4	193.1 ± 66.8	202.1 ± 70.3	224.2 ± 76.8
*p*	0.906 *		0.431 *		0.742 *		0.900 *		0.159 *	
SII (×10^9^/L)	3477.4 ± 2760.4	4024.6 ± 1829.4	2316.4 ± 1557.0	3154.0 ± 1717.5	1441.2 ± 1165.7	1872.9 ± 1059.9	1264.1 ± 703.3	2199.1 ± 1467.5	1314.5 ± 927.2	2416.5 ± 1664.5
*p*	0.342 *		0.034 *		0.126 *		<0.001 *		<0.001 *	
SIRI (×10^9^/L)	15.9 ± 12.5	18.8 ± 10.6	10.3 ± 9.8	14.7 ± 9.9	5.9 ± 5.7	8.8 ± 5.5	3.8 ± 2.7	8.4 ± 5.7	2.9 ± 2.8	6.7 ± 5.6
*p*	0.274 *		0.068 *		0.039 *		<0.001 *		<0.001 *	

G1—group without abscess development (*n* = 176), G2—group with an abscess development (*n* = 30), NLR—Neutrophil-to-Lymphocyte Ratio, NMR—Neutrophil-to-Monocyte Ratio, MLR—Monocyte-to-Lymphocyte Ratio, PLR—Platelet-to-Lymphocyte Ratio, SII—Systemic Immune-Inflammation Index, SIRI—Systemic Inflammatory Response Index, L—liter, * Student’s *t*-test.

**Table 6:** The average of individual percentage changes in variables of interest by groups (*n* = 206).

Variables	−1 ↔ 1st Day		1st ↔ 2nd/3rd Day		2nd/3rd ↔ 4th/5th Day		4th/5th ↔ 6th/7th Day	
	G1	G2	G1	G2	G1	G2	G1	G2
Leukocytes (×10^9^/L)	−24.2 ± 27.9%	−27.4 ± 23.2%	−16.1 ± 26.4%	−5.0 ± 29.0%	+2.4 ± 36.0%	+7.6 ± 42.4%	+5.1 ± 39.9%	+4.1 ± 34.9%
*p*	0.568 *		0.053 *		0.515 *		0.904 *	
Neutrophils (×10^9^/L)	−23.6 ± 32.6%	−23.3 ± 30.6%	−22.1 ± 36.5%	−20.9 ± 26.6%	−6.9 ± 38.9%	+11.1 ± 59.3%	+4.6 ± 57.4%	+8.0 ± 42.8%
*p*	0.875 *		0.895 *		0.114 *		0.397 *	
Lymphocytes (×10^9^/L)	+4.9 ± 73.1%	+3.5 ± 46.7%	+30.7 ± 47.6%	+42.9 ± 51.2%	+30.9 ± 44.5%	+21.9 ± 42.1%	+25.7 ± 45.5%	+16.0 ± 33.5%
*p*	0.939 *		0.317 *		0.412 *		0.353 *	
Monocytes (×10^9^/L)	−13.7 ± 32.0%	−8.3 ± 32.4%	+3.1 ± 62.7%	+6.9 ± 32.2%	+2.6 ± 39.0%	+4.9 ± 33.0%	−3.9 ± 41.9%	−5.7 ± 28.2%
*p*	0.525 *		0.795 *		0.802 *		0.850 *	
NLR	+3.8 ± 71.3%	−5.4 ± 76.4%	−36.0 ± 34.7%	−35.1 ± 40.5%	−22.2 ± 39.8%	+6.0 ± 79.2%	−14.8 ± 41.5%	−0.2 ± 44.9%
*p*	0.635 *		0.921 *		0.034 *		0.150 *	
NMR	−4.7 ± 46.9%	−10.2 ± 46.5%	−11.8 ± 55.2%	−25.2 ± 16.6%	−5.1 ± 32.7%	+6.2 ± 49.8%	+11.5 ± 37.2%	+14.1 ± 27.4%
*p*	0.653 *		0.290 *		0.237 *		0.759 *	
MLR	+6.0 ± 54.9%	+3.4 ± 44.8%	−15.6 ± 41.4%	−12.5 ± 52.3%	−17.5 ± 30.7%	−6.0 ± 38.1%	−12.8 ± 65.5%	−11.1 ± 38.7%
*p*	0.850 *		0.781 *		0.164 *		0.907 *	
PLR	+10.7 ± 66.8%	+10.5 ± 54.6%	−4.4 ± 45.5%	−18.3 ± 37.8%	+8.9 ± 38.1%	+20.3 ± 24.4%	+19.3 ± 48.6%	+26.3 ± 45.7%
*p*	0.994 *		0.214 *		0.231 *		0.548 *	
SII (×10^9^/L)	−10.7 ± 73.3%	−11.8 ± 72.0%	−31.7 ± 35.9%	−32.7 ± 48.9%	+5.4 ± 64.5%	+37.3 ± 86.4%	+21.7 ± 80.6%	+31.8 ± 55.0%
*p*	0.954 *		0.921 *		0.078 *		0.121 *	
SIRI (×10^9^/L)	−12.2 ± 66.8%	−18.1 ± 54.3%	−36.2 ± 45.2%	−23.0 ± 78.3%	−18.4 ± 51.5%	+16.3 ± 98.3%	−3.1 ± 111.2%	+0.8 ± 71.9%
*p*	0.733 *		0.334 *		0.039 *		0.176 *	
CRP (mg/L)	+148.9 ± 446.6%	+108.1 ± 241.0%	−7.8 ± 64.7%	−12.7 ± 29.1%	−40.8 ± 27.1%	−0.5 ± 61.8%	−62.8 ± 38.4%	−39.4 ± 39.6%
*p*	0.290 *		0.692 *		0.001 *		<0.001 *	

G1—group without abscess development (*n* = 176), G2—group with an abscess development (*n* = 30), NLR—Neutrophil-to-Lymphocyte Ratio, NMR—Neutrophil-to-Monocyte Ratio, MLR—Monocyte-to-Lymphocyte Ratio, PLR—Platelet-to-Lymphocyte Ratio, SII—Systemic Immune-Inflammation Index, SIRI—Systemic Inflammatory Response Index, CRP—C-reactive protein, L—liter, * Student’s *t*-test.

**Table 7:** Multivariable logistic regression analysis.

Variable	Adjusted OR	SE	z	*p*-Value	Lower Bound (95% CI)	Upper Bound (95% CI)
Rebound tenderness	1.38	0.444	0.73	0.468	0.58	3.30
Leukocytes (×10^9^/L)	1.03	0.037	0.84	0.399	0.96	1.11
Neutrophils (%)	1.10	0.043	2.12	0.034	1.01	1.19

OR is the adjusted odds ratio from multivariable logistic regression, SE is the standard error of the regression coefficient, z is the Wald statistic obtained as the coefficient divided by its standard error, and CI is the confidence interval.

## Data Availability

The data that support the findings of this study are available upon request from the corresponding author.

## Abbreviations

The following abbreviations are used in this manuscript:

SIRS	Systemic inflammatory response syndrome
CT	Computed tomography
RUQ	Right upper quadrant
RLQ	Right lower quadrant
LUQ	Left upper quadrant
LLQ	Left lower quadrant
US	Ultrasound
LOS	Length of stay
ICU	Intensive care unit
AUC	Area Under the Curve
ROC	Receiver Operating Characteristic
MCV	Mean corpuscular volume
MCH	Mean corpuscular hemoglobin
MCHC	Mean corpuscular hemoglobin concentration
RDW-CV	Red cell distribution width—coefficient of variation
MPV	Mean platelet volume
IG	Immature granulocytes
CRP	C-reactive protein
NLR	Neutrophil-to-Lymphocyte Ratio
NMR	Neutrophil-to-Monocyte Ratio
MLR	Monocyte-to-Lymphocyte Ratio
PLR	Platelet-to-Lymphocyte Ratio
SII	Systemic Immune-Inflammation Index
SIRI	Systemic Inflammatory Response Index
WSES	World Society of Emergency Surgery
